# Percutaneous and endoscopic combined treatment of bladder and renal lithiasis in mitrofanoff conduit

**DOI:** 10.1590/S1677-5538.IBJU.2021.0815

**Published:** 2022-02-22

**Authors:** Raffaele Inzillo, Jean Emmanuel Kwe, Elisa Simonetti, Riccardo Milandri, Marco Grande, Davide Campobasso, Stefania Ferretti, Bernardo Rocco, Salvatore Micali, Antonio Frattini

**Affiliations:** 1 Guastalla Hospital Department of Urology Emilia-Romagna Italy Department of Urology, Guastalla Hospital, Emilia-Romagna, Italy; 2 University of Modena and Reggio Emilia Department of Urology Italy Department of Urology, University of Modena and Reggio Emilia, Italy; 3 Parma Hospital Department of Urology Parma Italy Department of Urology, Parma Hospital, Parma, Italy

## Abstract

**Introduction and Objectives:**

Treatment of bulky lithiasis in continent and non-continent urine storage reservoirs has been widely described and debated (1). Less is known about the optimal treatment in patients with a Mitrofanoff conduit. If voiding in these patients is incomplete, leading to recurrent symptomatic bacteriuria, formation of large lithiasis can be a common long-term complication (2, 3).

**Materials and Methods:**

This video describes a 19-year-old woman who underwent major open surgery at the age of six, with the configuration of a continent intestinal reservoir with a Mitrofanoff conduit. In 2020, she was referred to our center with a large stone in the reservoir and a minor stone in the inferior left renal calyx.

We decided to proceed using a percutaneous approach with an “endovision technique” puncture for the bladder stone, combined with a retrograde intrarenal surgery for the renal stone. The MIP System “M size” was used to perform the percutaneous procedure, thus allowing a single-step dilation. The puncture and the dilation were followed endoscopically with a flexible ureterorenoscope avoiding the use of x-rays.

The procedure was carried out as follows. The first step consisted in the insertion of a hydrophilic guidewire through the Mitrofanoff conduit. A flexible ureterorenoscope was then inserted coaxial to the guidewire. The percutaneous puncture, using an 80G needle, was followed endoscopically. Two guidewires were inserted, the first as a safety guidewire and the second for the tract dilation. The “single-step” dilation technique using the MIP system was performed and followed endoscopically. For the bladder lithotripsy, a dual-action lithotripter that combines ultrasonic and mechanical energy was used. Finally, a flexible ureterorenoscope and a basket for the retrieval of a single inferior caliceal stone were used. The procedure ended after positioning a single J stent in the left kidney and a nephrostomy tube in the reservoir.

**Results:**

The operative time was 80 minutes and the fluoroscopy time was 6 seconds. Hemoglobin and creatinine serum levels remained stable after the procedure and the patient was discharged on the third post-operative day, after removing both the single J and the nephrostomy tube. Follow-up lasted 12 months, with no bladder or renal stone recurrence, maintaining good continence of the Mitrofanoff conduit.

**Conclusion:**

In patients who have undergone several major surgeries a mini-invasive approach is advisable, not only for the morbidity of an open approach, but also for the increased risk of complications while handling an intestinal reservoir. Regarding a pure endoscopic approach, the passage of a nephroscope or a cystoscope through the Mitrofanoff conduit, combined with the continuous traction during the lithotripsy, could damage and compromise its continence. For this reason, the percutaneous approach is the most suitable method in these specific and rare cases.
